# Effect of sodium-glucose cotransporter-2 inhibitors on blood pressure in patients with heart failure: a systematic review and meta-analysis

**DOI:** 10.1186/s12933-022-01574-w

**Published:** 2022-07-25

**Authors:** Min Li, Tieci Yi, Fangfang Fan, Lin Qiu, Zhi Wang, Haoyu Weng, Wei Ma, Yan Zhang, Yong Huo

**Affiliations:** 1 Department of Cardiovascular Disease, Peking University First Hospital, Beijing, China; 2 Echocardiography Core Lab, Institute of Cardiovascular Disease at Peking University First Hospital, Beijing, China; 3 Hypertension Precision Diagnosis and Treatment Research Center, Peking University First Hospital, Beijing, China; 4 Key Laboratory of Molecular Cardiovascular Sciences (Peking University), Ministry of Education, Beijing, China; 5 Division of Cardiology, Peking University First Hospital, Dahongluochang Street, Xicheng District, Beijing, 100034 China

**Keywords:** Heart failure, Sodium-glucose cotransporter-2 inhibitors, Blood pressure, Meta-analysis

## Abstract

**Background:**

Recent studies have shown that sodium-glucose cotransporter-2 inhibitors (SGLT2i) can achieve significant improvement in blood pressure in people with diabetes. Furthermore, randomized controlled trials (RCTs) have established that SGLT2i have a cardioprotective effect in adults with heart failure (HF). Therefore, we performed this systematic review an meta-analysis to determine the effect of SGLT2i on blood pressure in patients with HF.

**Methods:**

We used the Medline, Cochrane Library, Embase, and PubMed databases to identify RCTs (published through to April 29, 2022) that evaluated the effect of SGLT2i on HF. The primary endpoint was defined as change in blood pressure. Secondary composite outcomes were heart rate, hematocrit, body weight, and glycated hemoglobin. The N-terminal pro-brain natriuretic peptide level, Kansas City Cardiomyopathy Questionnaire scores, and estimated glomerular filtration rate were also evaluated.

**Results:**

After a literature search and detailed evaluation, 16 RCTs were included in the quantitative analysis. Pooled analyses showed that SGLT2i were associated with a statistically significant reduction in systolic blood pressure of 1.68 mmHg (95% confidence interval [CI] − 2.7, − 0.66; P = 0.001; I^2^ = 45%) but not diastolic blood pressure (mean difference [MD] −1.06 mmHg; 95% CI −3.20, 1.08; P = 0.33; I^2^ = 43%) in comparison with controls. Furthermore, SGLT2i decreased body weight (MD − 1.36 kg, 95% CI − 1.68, − 1.03; P < 0.001; I^2^ = 61%) and the glycated hemoglobin level (MD − 0.16%, 95% CI − 0.28, −0.04, P = 0.007; I^2^ = 91%) but increased hematocrit (MD 1.63%, 95% CI 0.63, 2.62, P = 0.001; I^2^ = 100%). There was no significant between-group difference in heart rate (MD − 0.35; 95% CI − 2.05, 1.35, P = 0.69; I^2^ = 0).

**Conclusions:**

SGLT2i decreased systolic blood pressure in patients with HF but had no effect on diastolic blood pressure. These inhibitors may have numerous potentially beneficial clinical effects in patients with HF.

**Supplementary Information:**

The online version contains supplementary material available at 10.1186/s12933-022-01574-w.

## Introduction

Although there has been considerable progress in the treatment of heart failure (HF) in recent years, HF-related morbidity and mortality remain high. The incidence of HF in Europe is currently approximately 3/1000 person-years in all age groups and approximately 5/1000 person-years in adults, and the true prevalence is likely to be higher [1]. In a cohort study, 1-year and 5-year mortality rates after diagnosis were 20% and 53%, respectively, regardless of type of HF [2].

Sodium-glucose cotransporter-2 inhibitors (SGLT2i) are novel agents that can improve the clinical outcomes in patients with HF [3, 4]. Although not indicated as antihypertensive agents, it has been found that treatment with SGLT2i is associated with sustained lowering of systolic blood pressure (SBP) and diastolic blood pressure (DBP) by 4–6 mmHg and 1–2 mmHg, respectively [5]. A more recent meta-analysis of 67 trials demonstrated a significant reduction in SBP with SGLT2i in patients with diabetes (mean difference [MD] − 2.89 mmHg; 95% confidence interval [CI] − 3.37, − 2.40) and DBP (MD − 1.44 mmHg; 95% CI − 1.68, − 1.20) [6]. Because 24-h ambulatory blood pressure (BP) is a better predictor of cardiovascular risk and mortality, a meta-analysis of six studies that used ambulatory BP monitoring was performed that suggested a 24-h reduction in ambulatory SBP by − 3.76 mmHg and in ambulatory DBP by − 1.83 mmHg [7]. Another meta-analysis in patients without diabetes demonstrated a mean reduction in SBP of − 1.90 mmHg (95% CI − 3.69, − 0.11) without any significant change in DBP (MD 0.27; 95% CI − 1.21, 1.76) [8].

There is a paradoxical relationship between BP and HF [9], and reducing SBP is thought to be the most beneficial treatment for HF. However, it has been reported that for each 10-mmHg decrease in SBP, there is an 18% increase in the risk of death, and that patients with lower SBP have worse outcomes than those with higher SBP [10, 11]. To date, there has been no meta-analysis of studies that have examined how SGLT2i influence BP in patients with HF. Therefore, we performed this systematic review and meta­analysis to determine the effect of SGLT2i on BP in patients with HF.

## Methods

This meta-analysis conformed to the standard guidelines, was written in accordance with the Preferred Reporting Items for Systematic Reviews and Meta-Analyses statement [12], and is registered in PROSPERO (CRD42022332279).

### Data sources and searches

We searched the PubMed, Medline, Cochrane Library, and Embase databases from inception to April 29, 2022. The search was divided into three concept groups. One group encompassed the terminology used to describe “sodium-glucose cotransporter-2 inhibitors,” another covered the terms relevant to “heart failure,” and the third addressed “randomized controlled trials (RCTs).” Medical Subject Headings and equivalent controlled vocabulary and keywords were used in each database. We screened the reference lists of eligible studies and systematic reviews and sought expert content input to identify additional eligible studies.

### Eligibility criteria

Only English-language publications were included. Studies were eligible for inclusion in the meta-analysis if they met the following criteria: (1) an RCT in humans; (2) adults with chronic HF who were treated with SGLT2i and compared with either a placebo group or an active control group; and (3) reporting of data on changes in BP from baseline in a form suitable for pooling. The premise of our study required that all study participants were patients with HF. Therefore, we only included RCTs with a HF prevalence of 100% regardless of whether or not they included other diseases.

### Outcomes

The primary endpoint was defined as change in BP. Secondary composite outcomes were HR, hematocrit, body weight, and glycated hemoglobin (HbA_1c_). The N-terminal pro-brain natriuretic peptide (NT-ProBNP) level, Kansas City Cardiomyopathy Questionnaire (KCCQ) scores, and estimated glomerular filtration rate (eGFR) were also evaluated because they were reported in the RCTs. Adverse events were defined as any serious adverse events reported in the studies.

### Data extraction and critical appraisal

The data were extracted by two authors working independently using a predefined, standardized protocol and data collection instrument. Information was recorded on study design, demographic characteristics, BP values, antihyperglycemic therapies, and serious adverse events reported in the trials. Any discrepancies were resolved by consensus among the authors.

### Risk of bias assessment

We evaluated the risk of bias using the revised Cochrane Risk of Bias Tool [13]. This tool has five domains (i.e., randomization process, deviations from intended interventions, missing outcome data, measurement of the outcome, and selection of the reported results) and provides an overall score. Each domain can be judged as low risk of bias, some concerns, and high risk of bias.

### Statistical analysis

Continuous variables are reported as the mean ± standard deviation or as the median (interquartile range). Numerical data are shown as the number (percentage). The meta-analysis was performed using a random-effects model with the inverse variance method. The standard deviation was calculated according to the Cochrane Handbook for Systematic Reviews of Interventions [14]. We detected the presence of statistical heterogeneity using the Cochrane P-value (significant when P < 0.10) and assessed the degree of heterogeneity using the I^2^ statistic (considered substantial when > 50%) [15]. To detect publication bias, we visually examined the funnel plots for SBP and assessed asymmetry using the Egger regression asymmetry test. To test the stability of our meta-analysis further, we performed multiple subgroup analyses according to baseline anti-HF agents, baseline characteristics, and type of studies.

We also performed a meta-regression analysis to determine if there was a significant linear association between BP reduction and cardiovascular death or hospitalizations for HF among those treated with SGLT2i. Furthermore, to evaluate the influence of each study on the overall effect size, a sensitivity analysis was conducted using the leave-1-out method (removing one study at a time and repeating the analysis) [16].

The statistical analyses were performed using the Revman software package (Review Manager, Version 5.1; The Cochrane Collaboration, Oxford, UK) and Stata software 12.0 (Stata Corp, College Station, TX, USA). All tests were 2-tailed and a P-value < 0.05 was considered statistically significant.

### Recommendations

We used the GRADE approach (Grading of RecommendationsAssessment, Development and Evaluation) to rate the quality of evidence of the pooled outcomes. The domains of assessment are statistical inconsistency, publication bias, risk of bias, indirectness, and statistical imprecision. The quality ratings are very low, low, moderate, and high [17].

## Results

### Study selection and characteristics

**Fig. 1:**
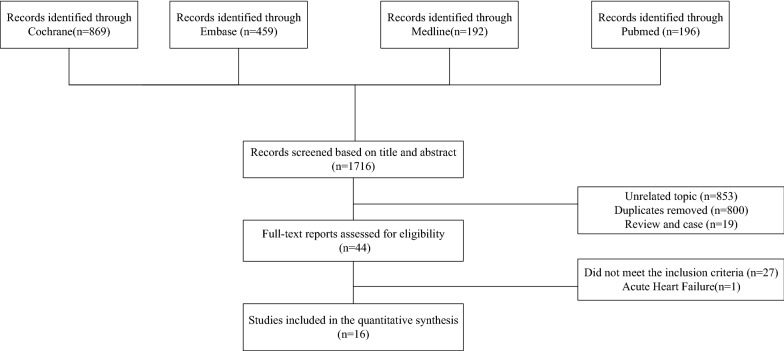
Flow diagram showing the study selection process

The literature search process and its results are shown in Fig. 1. We identified 1716 potentially relevant publications. After screening and removal of duplicates, we selected 44 articles for full-text screening. Finally, 16 RCTs were included in the analysis. Of these, one study was a cross-over RCT [18], four evaluated dapagliflozin [3, 19–21], seven assessed empagliflozin [4, 18, 22–26], three evaluated canagliflozin [27–29], one investigated luseogliflozin [30], one study included 5 treatment arms (empagliflozin, licogliflozin [2.5 mg, 10 mg, and 50 mg], and placebo). We used the empagliflozin and placebo arms of this RCT to retain homogeneity in the intervention; the licogliflozin arm was not included because licogliflozin is an SGLT1/2i and not an SGLT2i [31]. In the included studies, dapagliflozin was used at a dose of 10 mg/day, empagliflozin at 10–25 mg/day, canagliflozin at 100 mg/day, and luseogliflozin at 2.5 mg/day. The intervention sample size ranged from 23 to 5988 patients and the follow-up duration was from 6 weeks to 26.2 months. Eight RCTs [3, 4, 18, 19, 24–26, 28] included adults who had HF with reduced ejection fraction (HFrEF) and four [21, 22, 29, 30] included patients who had HF with preserved ejection fraction (HFpEF), The other four studies included participants with any type of HF regardless of left ventricular ejection fraction (LVEF) [20, 23, 27, 31]. The LVEF cut-off value used to determine HFrEF was 40% in six studies [3, 4, 19, 24, 26, 28] and 50% in two studies [18, 25] and that for HFpEF was 50% in one study [29], 40% in one study [22], and 45% in two studies [21, 30]. The majority of studies required patients to receive standard treatment for HF. Key clinical data for each of the included studies are provided in Table 1 and Additional file 6: Table S1.

**Table 1:** Characteristics of included studies

Trial or Author	Time	Design	SGLT2i	Control	Number (I/C)	Follow up time	LVEF
DAPA-HF [3]	2019	RCT	DAPA 10 mg once daily	Placebo	2373/2371	18.2 months	LVEF ≤ 40%
DEFINE-HF [19]	2019	RCT	DAPA 10 mg once daily	placebo	131/132	12 Weeks	LVEF ≤ 40%
De Boer [31]	2019	RCT	Licogliflozin (2.5 mg, 10 mg, 50 mg) EMPA 25 mg once daily	Placebo	91/33	12 Weeks	NR
MUSCAT‐HF [30]	2019	open-label RCT	Luseogliflozin 2.5 mg once daily	voglibose 0.2 mg Three times a day	83/82	12 Weeks	LVEF ≥ 45%
REFORM [20]	2019	RCT	DAPA 10 mg once daily	placebo	28/28	1 year	NR
CANDLE [27]	2020	open-label RCT	CANA 100 mg once daily	Glimepiride 0.5 mg once daily	113/120	24 Weeks	NR
CANA-HF [28]	2020	RCT	CANA 100 mg once daily	sitagliptin 100 mg once daily	17/19	12 Weeks	LVEF ≤ 40%
EMPEROR-Reduced [4]	2020	RCT	EMPA 10 mg once daily	Placebo	1863/1867	16 months	LVEF ≤ 40%
EMPEROR-Preserved [22]	2021	RCT	EMPA 10 mg once daily	placebo	2997/2991	26.2 months	LVEF > 40%
EMBRACE-HF [23]	2020	RCT	EMPA 10 mg once daily	placebo	33/32	12 Weeks	NR
RECEDE-CHF [18]	2020	cross-over RCT	EMPA 25 mg once daily	Placebo	12/11	6 Weeks	LVEF < 50%
CANONICAL [29]	2021	open-label RCT	CANA 100 mg once daily	NR	42/40	24 Weeks	LVEF ≥ 50%
Empire HF [24]	2021	RCT	EMPA 10 mg once daily	placebo	95/95	12 Weeks	LVEF ≤ 40%
PRESERVED-HF [21]	2021	RCT	DAPA 10 mg once daily	placebo	162/162	12 Weeks	LVEF ≥ 45%
Pietschner [25]	2021	RCT	EMPA 10 mg once daily	placebo	36/17	12 Weeks	LVEF < 50%
SUGAR-DM-HF [26]	2021	RCT	EMPA 10 mg once daily	placebo	52/53	36 Weeks	LVEF ≤ 40%

*CANA* canagliflozin, *DAPA* dapagliflozin, *EMPA* empagliflozin, *I/C* intervention/control, *LVEF* left ventricular ejection fraction, *NR* not reported, *RCT* randomized controlled trial, *SGLT2i* sodium-glucose cotransporter-2 inhibitors

### Risk of bias assessment

The overall risk of bias was judged to be low in 12 of the 16 RCTs [3, 4, 18–26, 28]. One study presented some concerns in terms of the reporting of results [31]. Three trials [27, 29, 30] were at high risk of bias because of their open-label design. Additional file 1: Figure S1 summarizes the outcome definitions for each trial.

### Primary outcome

**Fig. 2:**
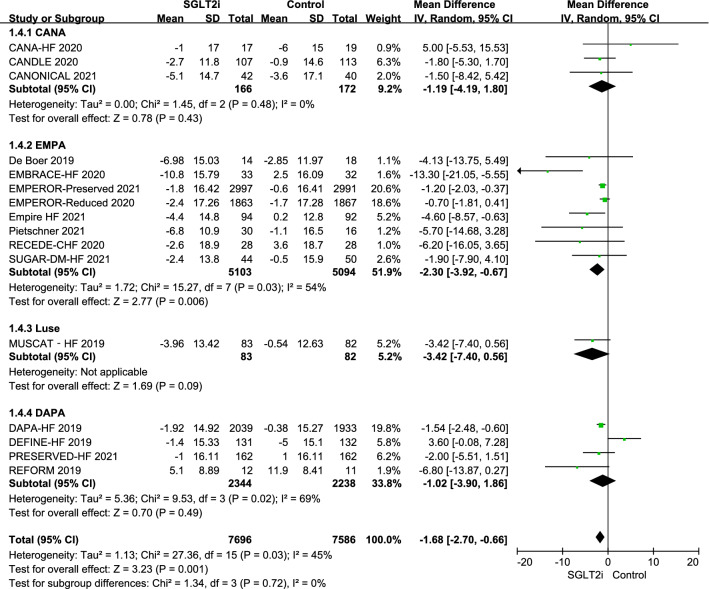
Effects of different types of sodium-glucose cotransporter-2 inhibitors on systolic blood pressure. *CI* confidence interval, *CANA* canagliflozin, *DAPA* dapagliflozin, *EMPA* empagliflozin, *IV* inverse variance, *Luse* luseogliflozin, *SD* standard deviation, *SGLT2i* sodium-glucose cotransporter-2 inhibitors

**Fig. 3:**
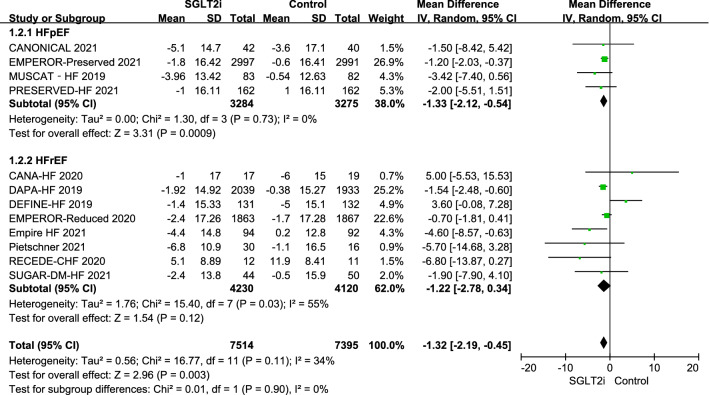
Effect of sodium-glucose cotransporter-2 inhibitors on systolic blood pressure according to left ventricular ejection fraction status. *HFrEF* heart failure with reduced ejection fraction, *HFpEF* heart failure with preserved ejection fraction, *LVEF* left ventricular ejection fraction

When the results of the 16 RCTs were pooled, use of an SGLT2i was associated with a statistically significant reduction in SBP from baseline of 1.68 mmHg (95% CI − 2.70, − 0.66; P = 0.001; I^2^ = 45%) in comparison with the control value. The mean reductions in SBP with canagliflozin, empagliflozin, luseogliflozin, and dapagliflozin were − 1.19 mmHg (95% CI − 4.19, 1.80), − 2.30 mmHg (95% CI − 3.92, − 0.67), − 3.42 mmHg (95% CI − 7.40, 0.56), and − 1.02 mmHg (95% CI − 3.90, 1.86), respectively. There was no significant difference in change in SBP according to type of SGLT2i used (P = 0.72). When grouped according to LVEF, use of SGLT2i in patients with HFpEF was associated with a reduction in SBP of 1.33 mmHg (95% CI − 2.12, − 0.54; P < 0.001; I^2^ = 0); however, there was no statistically significant difference between the groups (P = 0.90) (Figs. 2 and 3).

**Fig. 4:**
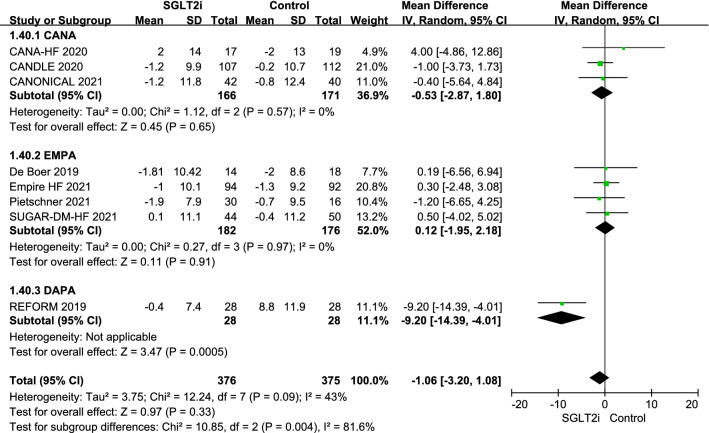
Effect of sodium-glucose cotransporter-2 inhibitors on diastolic blood pressure. *CI* confidence interval, *CANA* canagliflozin, *DAPA* dapagliflozin, *EMPA* empagliflozin, *IV* inverse variance, *Luse* luseogliflozin, *SD* standard deviation, *SGLT2i* sodium-glucose cotransporter-2 inhibitors

Only eight of the 16 RCTs reported data for DBP [20, 24–29, 31]. There was no statistically significant difference in DBP between the SGLT2i and control groups (MD − 1.06 mmHg; 95% CI − 3.20, 1.08; P = 0.33; I^2^ = 43%). The mean differences observed with canagliflozin, empagliflozin, and dapagliflozin were − 0.53 mmHg (95% CI − 2.87, 1.80), 0.12 mmHg (95% CI − 1.95, 2.18), and − 9.20 mmHg (95% CI − 14.39, − 4.01), respectively. There was a significant difference (P = 0.004) according to type of SGLT2i used (Fig. 4).

### Secondary outcomes and subgroups

**Fig. 5:**
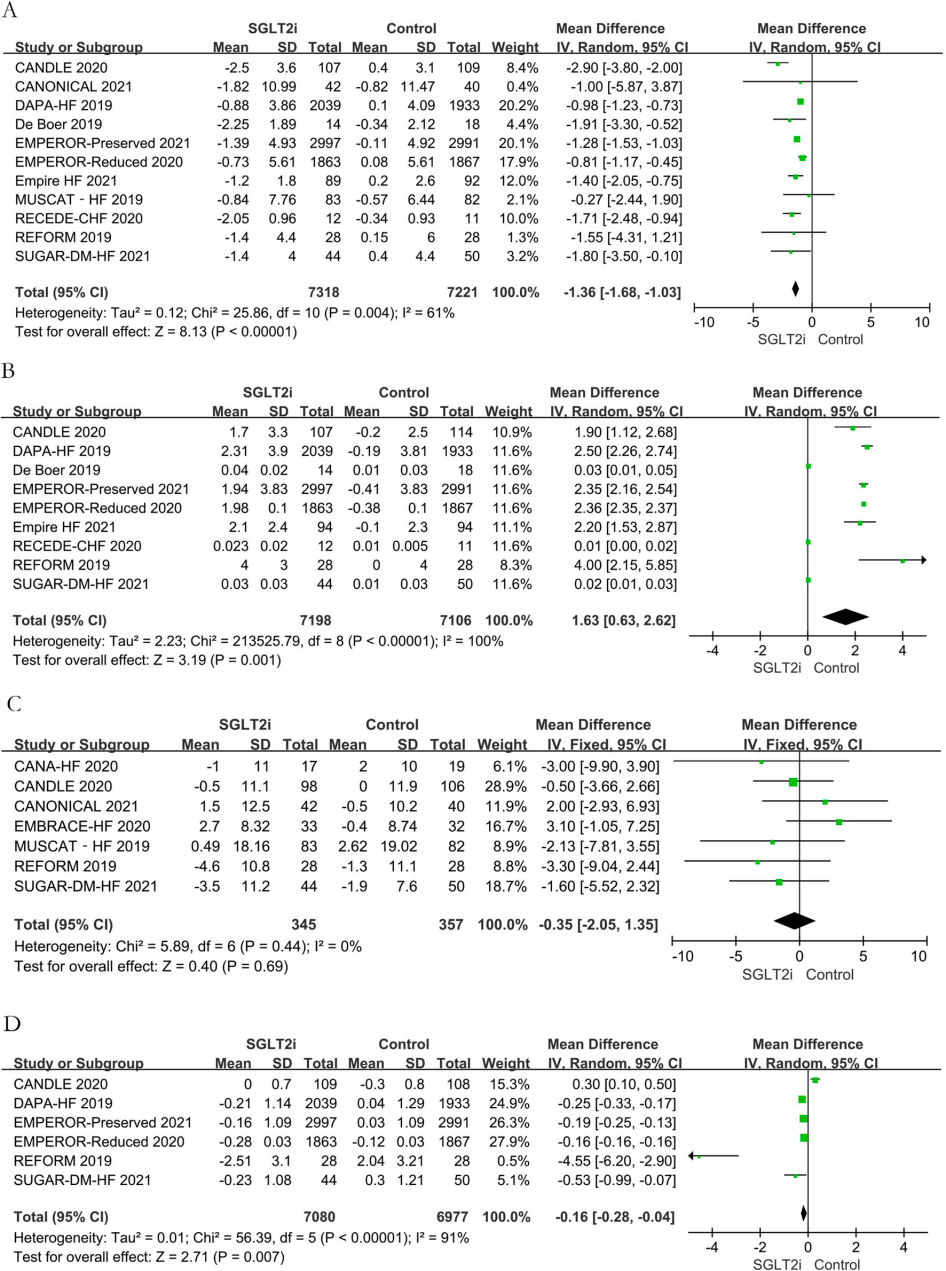
Effects of sodium-glucose cotransporter-2 inhibitors on secondary outcomes. **A**: Body weight. **B**: Hematocrit; **C**: Heart rate. **D**: Glycated hemoglobin. *CI* confidence interval, *SD* standard deviation, *SGLT2i* sodium-glucose cotransporter-2 inhibitors

Meta-analysis showed that SGLT2i reduced body weight (MD − 1.36 kg; 95% CI − 1.68, − 1.03; P < 0.001; I^2^ = 61%) but increased hematocrit (MD 1.63**%**; 95% CI 0.63, 2.62; P = 0.001; I^2^ = 100%). There was no significant difference in HR (MD − 0 .35 bpm, 95% CI − 2 .05, 1.35; P = 0.69; I^2^ = 0) between the SGLT2i and control groups. Considering that the time during which hemoglobin is metabolically active is approximately 120 days, we only included studies with a follow-up duration of  ≥ 12 weeks in the meta-analysis when evaluating HbA_1c_. The results suggested that SGLT2i can significantly reduce the HbA_1c_ level (MD − 0.16%, 95% CI − 0.28, − 0.04, P = 0.007; I^2^ = 91%), as shown in Fig. 5. Furthermore, SGLT2i reduced the NT-proBNP level (MD -60.31 pg/mL; 95% CI − 105.43, − 15.20; P = 0.009; I^2^ = 77%) but increased the KCCQ score (MD 1.97; 95% CI 1.16, 2.77, P < 0.001; I^2^ = 29%). There was no significant difference in eGFR (MD 0.98 mL·min^− 1^·1.73 m^−2^; 95% CI − 0.20, 2.17, P = 0.10; I^2^ = 73%) between the SGLT2i and control groups (Additional file 2: Figure S2).

Subgroup analysis showed no significant association between SGLT2i and SBP according to baseline characteristics, baseline anti-HF agents used, or type of study. However, the antihypertensive effect of SGLT2i showed a strong downward trend, albeit not statistically significant, in the groups with larger decreases in HbA_1c_ and NT-proBNP (Table 2).

**Table 2:** Outcomes of Subgroup Analysis of SBP

Subgroup	Number	MD 95CI	P	I^2^	Subgroup	Number	MD 95CI	P	I^2^
Age (y)			0.46	0	ACEI /ARB (%)			0.30	5
≥ 70	3	− 1.30 (− 2.10, − 0.49)			≥ 80	8	− 1.23 (− 1.77, − 0.70)		
< 70	13	− 1.93 (− 3.40, − 0.45)			< 80	7	− 2.85 (− 5.91, 0.20)		
BMI (kg/m^2^)			0.59	0	β-block (%)			0.55	0
≥ 28	13	− 1.63 (− 2.80, − 0.46)			≥ 80	10	− 1.52 (− 2.83, − 0.21)		
< 28	3	− 2.38 (− 4.84, 0.08)			< 80	4	− 2.26 (− 4.27, − 0.24)		
Control			0.83	0	MRA (%)			0.10	63.3
Placebo	13	− 1.70 (− 2.87, − 0.53)			≥ 50	6	− 0.85 (− 2.39, 0.69)		
Other drugs	4	− 2.00 (− 4.39, 0.39)			< 50	8	− 2.97 (− 4.97, − 0.98)		
Time (weeks)			0.23	31.7	Diuretic (%)			0.22	33.3
> 12	8	− 1.25 (− 2.05, − 0.46)			≥ 80	5	− 1.51 (− 2.38, − 0.64)		
≤ 12	9	− 3.22 (− 6.40, − 0.04)			< 80	8	− 2.79 (− 4.66, − 0.93)		
Number			0.07	69.9	△NT-proBNP (pg/ml)			0.18	43.7
≥ 100	10	− 1.23 (− 1.96, − 0.51)			≥ 200	4	− 4.80 (− 10.05, 0.44)		
< 100	7	− 4.53 (− 7.95, − 1.10)			< 200	7	− 1.17 (− 2.20, − 0.13)		
Diabetes			0.33	9.6	△Weight (kg)			0.92	0
Yes	8	− 2.65 (− 4.68, − 0.63)			≥ 2	3	− 1.03 (− 6.26, 4.20)		
No matter with or without DM	9	− 1.46 (− 2.71, − 0.21)			< 2	8	− 1.31 (− 1.84, − 0.79)		
Hypertension (%)			0.66	0	△HbA1c (%)			0.12	58.0
≥ 80	4	− 1.26 (− 2.06, − 0.45)			≥ 0.3	6	− 3.95 (− 7.67, − 0.23)		
< 80	6	− 1.63 (− 3.08, − 0.18)			< 0.3	5	− 0.94 (− 1.82, − 0.05)		

*ACEI* angiotensin-converting enzyme inhibitor, *ARB* angiotensin II receptor blocker, *BMI* body mass index, *CI* confidence interval, *DM* diabetes mellitus, *HbA1c* glycated hemoglobin, *MD* mean difference, *NT-ProBNP* N-terminal pro-brain natriuretic peptide

For every 1-mmHg reduction in SBP, there was a statistically non-significant 8% relative risk reduction in cardiovascular death (odds ratio [OR] 0.92; 95% CI 0.53–1.58; P = 0.711). Furthermore, there was no apparent association between this reduction and hospitalizations for HF (OR 1.05; 95% CI 0.87–1.28; P = 0.570). The details are shown in Additional file 3: Figure S3.

### Serious adverse events

Nine studies reported occurrence of serious adverse events during the study period [3, 4, 19–23, 26, 31]. Pooling of the results of these studies showed that SGLT2i reduced the risk of any serious adverse events (OR 0.85; 95% CI 0.77, 0.93; I^2^ = 27%; P < 0.001). The details were shown in Additional file 4: Figure S4.

### Sensitivity analyses and publication bias

In the leave-1-out sensitivity analyses, the pooled effect estimates remained similar across all studies, confirming that the significant difference between the studied groups was the overall effect of all the included studies. The details are shown in Additional file 6: Table S2.

Although the funnel plotsfor SBP showed relative asymmetry, the Egger linear regression test confirmed the robustness of the SBP (t = − 1.75; 95% CI − 1.69, 0.17; P = 0.103). The data are provided in Additional file 5: Figure S5.

### GRADE summary of findings

On pooled analysis of outcomes, there was moderate certainty of evidence for the primary outcomes, including SBP and DBP. The secondary composite outcomes of body weight, hematocrit, HR, and HbA_1c_ had a moderate, low, high, and very low certainty of evidence, respectively (Additional file 6: Table S3).

## Discussion

In this meta­analysis of patients with HF, we found that SGLT2i significantly reduced SBP but had no effect on DBP. SGLT2i were also associated with a reduction in body weight and HbA_1c_ but with an increment in hematocrit. However, our findings should be interpreted in the context of a moderate level of heterogeneity.

Previous meta-analyses have shown that SGLT2i can significantly reduce BP in patients with diabetes [6, 32]. Many RCTs have shown that SGLT2i can also reduce BP in patients with HF [3, 30]. The DAPA-HF study showed that dapagliflozin can significantly decrease SBP [3] whereas the EMPEROR-Reduced trial did not find a statistically significant reduction [4]. Given the concern about hypotension that often leads to withholding of potentially beneficial therapy in patients with HF, it is important to evaluate the effect of SGLT2i on BP.

The exact mechanisms of the antihypertensive effect of SGLT2i have not been fully elucidated. SGLT2i have been noted to induce osmotic diuresis and natriuresis [33], improve arterial stiffness [34], reduce sympathetic activity [35], suppress the renal renin-angiotensin system [36], decrease oxidative stress, and potentially improve endothelial dysfunction [37]. These combined actions result in a significant reduction in BP.

A recent systematic review included RCTs and subgroup analyses enrolling HF patients randomized to a SGLT2i. However, there are some differences between their research and our present review and meta-analysis. Chambergo-Michilot et al. included nine RCTs that compared an SGLT2i with placebo in patients with HF, six of which reported SBP values and 2 reported DBP values. Finally showed that SGTL2i significantly reduced SBP (MD −0.70 mmHg; 95% CI −0.73, −0.68; I^2^ = 48%) but not DBP (MD −4.76 mmHg; 95% CI −13.95, 4.42; I^2^ = 79%) [38]. The findings of our present meta-analysis are consistent with this meta-analysis conducted by Chambergo-Michilot et al. However, we evaluated more RCTs and outcomes, and instead of repeating further analyses of existing meta-analyses, we expanded the pooled results by incorporating new RCTs so as to provide comprehensive data support. Teo et al. performed another meta-analysis that included ten RCTs of patients with HF and demonstrated no significant difference in the treatment effect on SBP [39]. However, the findings are not directly comparable because of differences in the eligibility criteria used. Despite the high number of new RCTs published, DAPA-HF [3], EMPEROR-Reduced [4], and EMPEROR-preserved [22] are the most significant trials because of their large sample size and inclusion of more than 3000 patients in each trial. Because of differences in the primary endpoints and a limited time for inclusion, Teo et al. did not include the studies with the most weight, resulting in inconsistent results. Furthermore, Teo et al. also included studies of patients with acute HF. Therefore, compared to the cited reviews, our findings are more homogenous in terms of the study population.

We also assessed the effect of SGLT2i in patients with HF according to ejection fraction. We found the reduction in SBP was pronounced in patients with HFpEF, despite no statistically significant difference between groups. The vast majority of patients with HFpEF have underlying hypertension [1], and lowering BP in hypertensive patients is the most important step in preventing HF [11]. However, a study in patients with HFrEF indicated that each 1-mmHg decrement in baseline SBP was associated with an approximately 4% increase in the risk of non-sudden cardiac death [40]. Therefore, changes in BP are particularly important in patients with HFrEF. A RCT have evidenced that dapagliflozin had a small effect on SBP in patients with HFrEF and was superior to placebo, even in individuals with SBP < 110 mmHg [41]. Our results are also consistent with this finding. Our meta-regression analysis also found that a change in SBP may not influence the risks of cardiovascular death and hospitalization in patients with HF. This finding may run parallel to the observations with angiotensin-converting enzyme inhibitors and beta-blockers, which have a certain effect on BP in patients with HF and can deliver significant survival benefit [40, 42].

Apart from SBP, the DBP level may also have a predictive value in patients with HF. One study found that elderly patients with HF and elevated DBP on admission had a lower risk of death at 30 days and 1 year [43]. Low DBP levels reduce coronary perfusion pressure, which can result in ischemia and myocardial damage and may lead to a poor prognosis in patients with HF [44]. We found that SGLT2i did not achieve a significant reduction in DBP; this will effectively ensure blood perfusion of the myocardium. On the basis of the results of our meta-analysis, we believe that changes in BP should probably not influence the decision to use or continue to use SGLT2i.

One clinical trial indicated that luseogliflozin can decrease HR effectively in patients with a higher HR at baseline [45]. Although we found no statistically significant change in our study, we still found a downward trend in HR. The decline in HR may be related to the ability of SGLT2i to reduce reflex sympathetic nerve hyperactivity and to influence other neurohormonal pathways [46]. Furthermore, another meta-analysis showed that use of SGLT2i was associated with a statistically significant increase in hematocrit from baseline (WMD 2.4%; 95% CI 2.2–2.6) in patients with type 2 diabetes [32]. We have reached the same conclusion in patients with HF. The results of the EMPA-REG OUTCOME trial indicated that changes in hematocrit, which are ostensibly a marker of the effects of the drug on volume, appeared to be an important mediator of the reduction in mortality risk [47]. Increased hematocrit might help to supply oxygen to peripheral tissues and mitigate HF-related symptoms.

The glucosuric effect of SGLT2i was consistently associated with a 2–3-kg lower body weight [48], which may have a beneficial impact on cardiovascular risk factors[49] and contribute to an overall reduction of BP. One study mentioned that weight loss of 2 kg contributed to a 28% overall reduction in SBP and a 24% overall reduction in DBP [50]. While loss of fluid may contribute to initial weight loss, the majority of steady-state weight loss that occurs on SGLT2i appears to result from loss of fat [48]. We found that SGLT2i can significantly reduce body weight, which is in line with previous findings. However, no correlation was shown between body weight and SBP in our subgroup analysis; the baseline SBP in patients with HF was low, and the amount of reduction was small, thereby weakening the relationship between body weight and SBP.

Our study also found that SGLT2i reduced the HbA_1c_ level in patients with HF, which is consistent with previous findings. A meta-analysis showed that dapagliflozin was associated with a reduction in HbA_1c_ (MD − 0.53%; 95% CI − 0.58, − 0.47; p < 0.001) [51]. HbA_1c_ was decreased by 1.08% (95% CI − 1.25, − 0.90; p < 0.001) in patients who received canagliflozin [52] and by 0.62% (95% CI − 0.68, − 0.57%) in those who received empagliflozin [53]. A secondary analysis of the randomized clinical trial known as TECOS showed U-shaped associations between HbA_1c_ and cardiovascular outcomes, with the lowest risk when HbA_1c_ was approximately 7%. Each one-unit increase above 7% was associated with hospitalization for HF [54]. Therefore, the reduction of HbA_1c_ was also beneficial for patients with HF.

Subgroup analysis showed no significant associations between SGLT2i and SBP in terms of baseline characteristics, baseline anti-HF drugs administered, or type of study. A meta-analysis indicated that SGLT2i improved cardiovascular outcomes and that the clinical benefit was comparable between diabetic and nondiabetic individuals, men and women, and younger and older patients with underlying HF [55]. Further research is needed to identify the most suitable populations for SGLT2i.

## Study limitations

This review and meta-analysis has several limitations. First, the majority of the included studies had relatively small sample sizes, potentially leading to unstable estimates of treatment effects given that smaller trials might be methodologically less robust and prone to report larger effect sizes. Second, the background therapies used were not uniform, which might explain some of the heterogeneity in our results. Third, the follow-up duration was short in most of the trials, and longer follow-up is required to assess the long-term effectiveness of SGLT2i. Finally, there was some moderate heterogeneity across the studies included in the meta-analysis; the results of the sensitivity analysis suggested that two studies, EMBRACE-HF [23] and DEFINE-HF [19], were the causes of this heterogeneity. EMBRACE-HF included patients with an elevated pulmonary artery diastolic pressure of ≥ 12 mmHg while those in DEFINE-HF had high rates of medical therapy as well as frequent use of devices. Therefore, baseline population characteristics may have been responsible for the heterogeneity. When these two studies were removed, the final conclusion was robust, namely, there was a significant reduction in SBP of 1.38 mmHg (95% CI −1.89, − 0.86; P < 0.001; I^2^ = 0%).

## Future directions

First, the effect of SGLT2i on SBP and DBP depends on the etiology of HF and the history of hypertension at baseline. Therefore, future trials must state the effect of SGLT2i on SBP and DBP according to the baseline BP level. This is particularly important in patients with a lower baseline BP. Second, the reduction in SBP was also associated with non-sudden cardiac death. Therefore, Except for cardiovascular and all-cause mortality, non-cardiac mortality should also be focused. Finally, further studies are also needed to confirm the safety and the difference between use of SGLT2i in patients with acute HF and those with HFrEF and HFpEF so as to provide a more comprehensive understanding of these agent.

## Conclusion

In our meta-analysis of 16 randomized controlled trials of SGLT2i in patients with HF, we demonstrated that these agents significantly reduce SBP but do not have an effect on DBP. Furthermore, SGLT2i improve metabolic parameters, including body weight and HbA_1c_, in patients with HF. These agents may have numerous potentially beneficial clinical effects in these patients.

## Supplementary Information

**Additional file 1: Figure S1.** Assessment of risk of bias in the included trials.

**Additional file 2: Figure S2. **Effect of sodium-glucose cotransporter-2 inhibitors on other outcomes. A: N-terminal pro-brain natriuretic peptide level. B: Kansas City Cardiomyopathy Questionnaire score. C: Estimated glomerular filtration rate.

**Additional file 3: Figure S3.** Meta-regression showing mean change in systolic blood pressure for cardiovascular death and hospitalizations for heart failure.

**Additional file 4: Figure S4.** Forest plot showing the difference in serious adverse events between the SGLT2i groups and the control groups. CI, confidence interval; SD, standard deviation; SGLT2i, sodium-glucose cotransporter-2 inhibitors.

**Additional file 5: Figure S5.** Funnel plot and Egger regression test for systolic blood pressure. SE, standard error; SMD, standard mean difference.

**Additional file 6: Table S1.** Baseline drugs and characteristics. **Table S2.** Sensitivity Analyses of the SGLT2i on SBP. **Table S3**. GRADE summary of findings.

## Data Availability

All datagenerated or analyzed during this study are included in published article.

## Abbreviations

DBP: Diastolic blood pressure
eGFR: Estimated glomerular filtration rate
HbA1c: Glycated hemoglobin
HF: Heart failure
HFpEF: HF with preserved ejection fraction
HFrEF: HF with reduced ejection fraction
HR: Heart rate
KCCQ: Kansas City Cardiomyopathy Questionnaire
LVEF: Left ventricular ejection fraction
NT-ProBNP: N-terminal pro-brain natriuretic peptide
RCT: Randomized controlled trials
SBP: Systolic blood pressure
SGLT2i: Sodium-glucose cotransporter-2 inhibitors
