# Supplementary material for: Effect of sodium-glucose cotransporter-2 inhibitors on blood pressure in patients with heart failure: a systematic review and meta-analysis
Source: Cardiovasc Diabetol. 2022 Jul 25;21:139. doi: 10.1186/s12933-022-01574-w (PMC9317067; doi:10.1186/s12933-022-01574-w)
Supplement: Supplementary file 1 — Additional file 1: Figure S1. Assessment of risk of bias in the included trials. [file 12933_2022_1574_MOESM1_ESM.pdf]

Study ID	Randomization process	Deviations from intended interventions	Missing outcome data	Measurement of the outcome	Selection of the reported result	Overall
DAPA-HF	+	+	+	+	+	+
CANA-HF 2020	+	+	+	+	+	+
CANDLE 2020	?	+	+	+	+	?
CANONICAL 2021	?	+	+	+	+	?
De Boer 2019	+	+	+	+	?	!
DEFINE-HF 2019	+	+	+	+	+	+
EMBRACE-HF 2020	+	+	+	+	+	+
EMPEROR-Preserved 2021	+	+	+	+	+	+
EMPEROR-Reduced 2020	+	+	+	+	+	+
Empire HF 2021	+	+	+	+	+	+
MUSCAT - HF 2019	?	+	+	+	+	?
Pietschner 2021	+	+	+	+	+	+
PRESERVED-HF 2021	+	+	+	+	+	+
RECEDE-CHF 2020	+	+	+	+	+	+
REFORM 2019	+	+	+	+	+	+
SUGAR-DM-HF 2021	+	+	+	+	+	+

+ Low risk
? Some concerns
? High risk
