# Supplementary figures and images for: Effect of sodium-glucose cotransporter-2 inhibitors on blood pressure in patients with heart failure: a systematic review and meta-analysis
Source: Cardiovasc Diabetol. 2022 Jul 25;21:139. doi: 10.1186/s12933-022-01574-w (PMC9317067; doi:10.1186/s12933-022-01574-w)

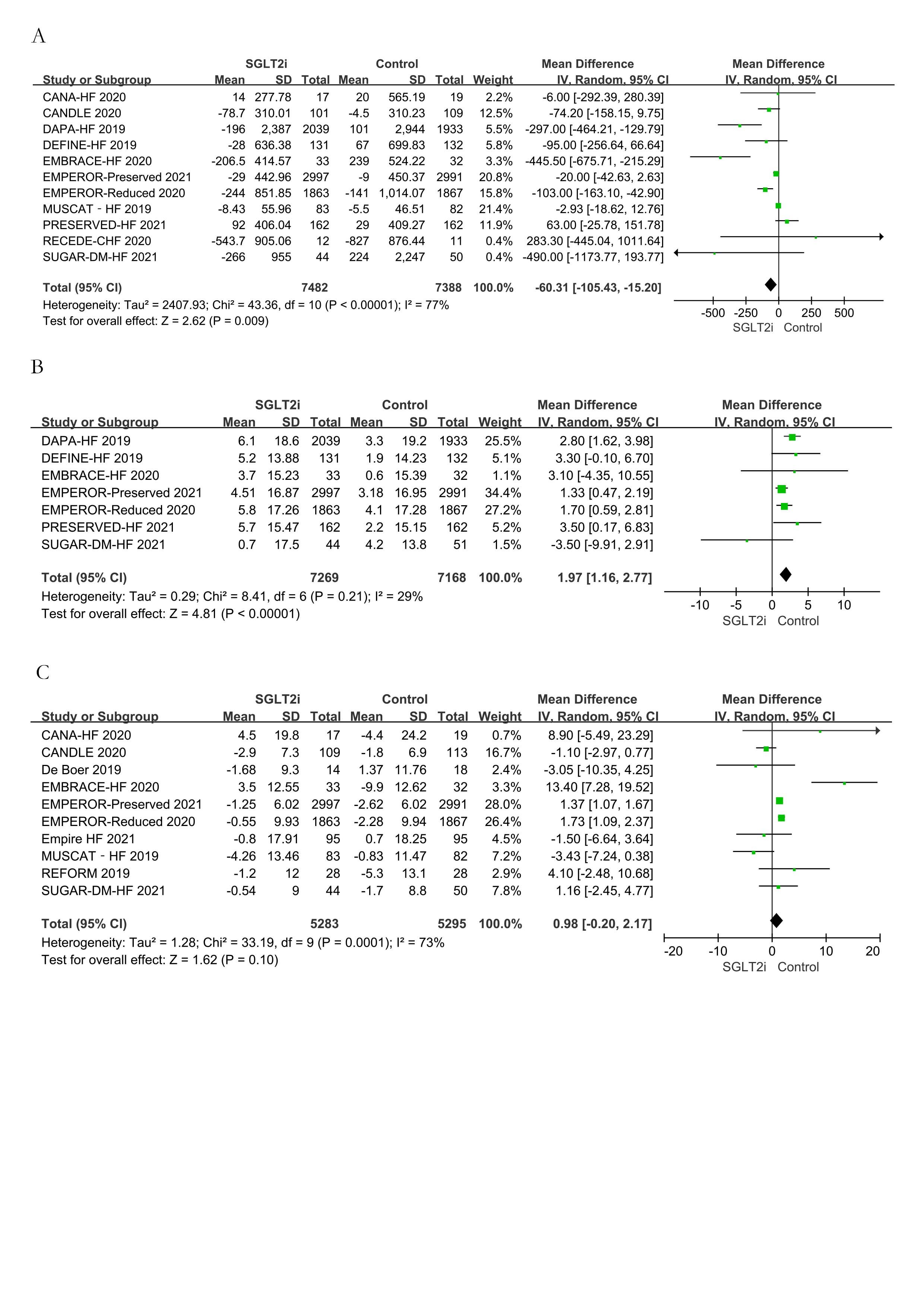

Supplement: Supplementary file 2 — Additional file 2: Figure S2. Effect of sodium-glucose cotransporter-2 inhibitors on other outcomes. A: N-terminal pro-brain natriuretic peptide level. B: Kansas City Cardiomyopathy Questionnaire score. C: Estimated glomerular filtration rate. [file 12933_2022_1574_MOESM2_ESM.jpg]

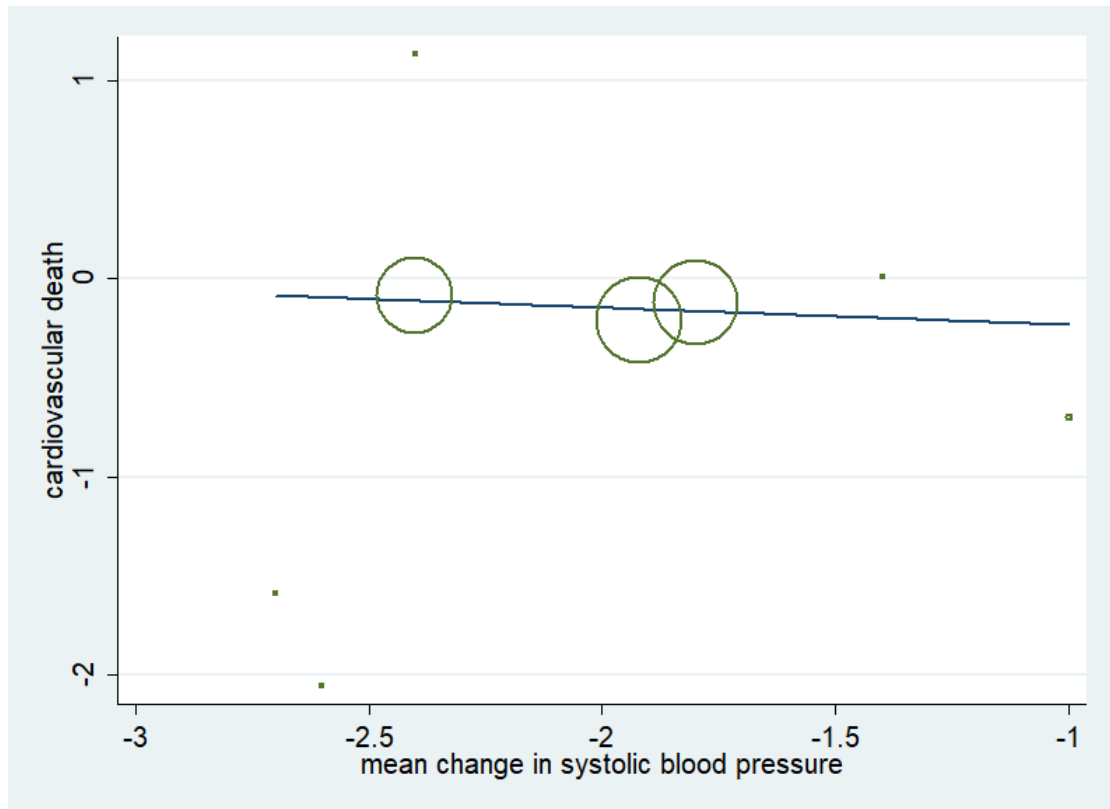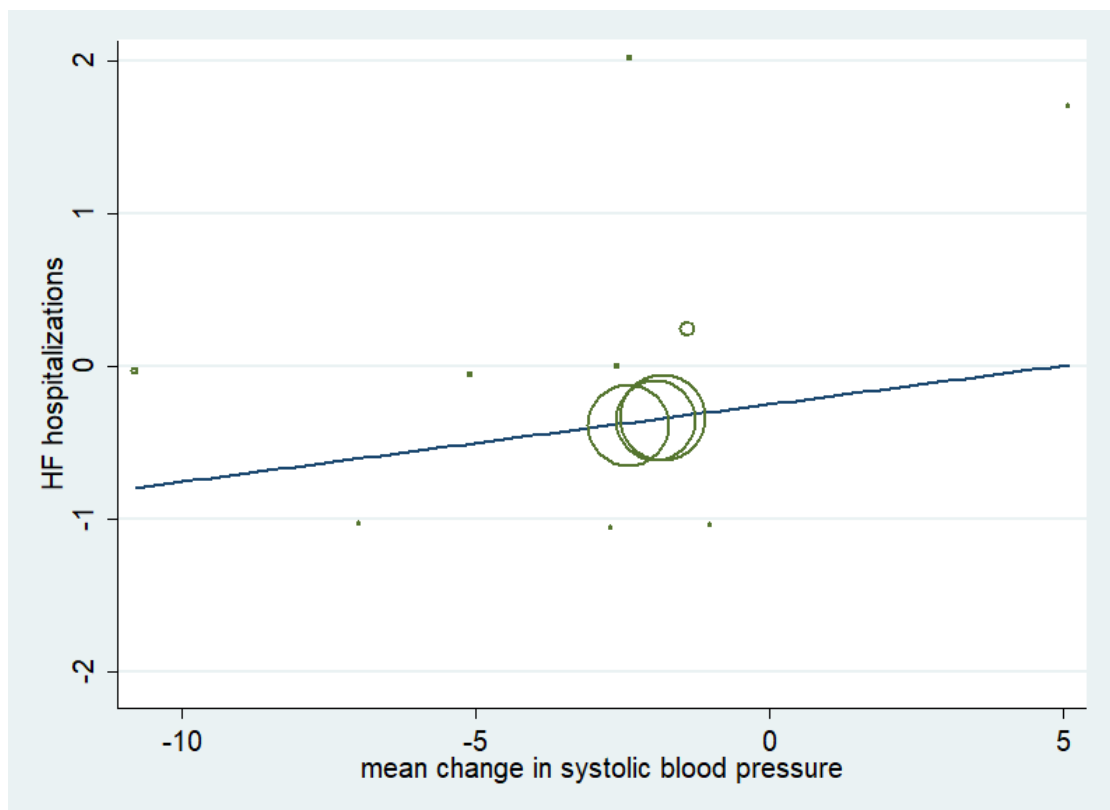

Supplement: Supplementary file 3 — Additional file 3: Figure S3. Meta-regression showing mean change in systolic blood pressure for cardiovascular death and hospitalizations for heart failure. [file 12933_2022_1574_MOESM3_ESM.pdf]

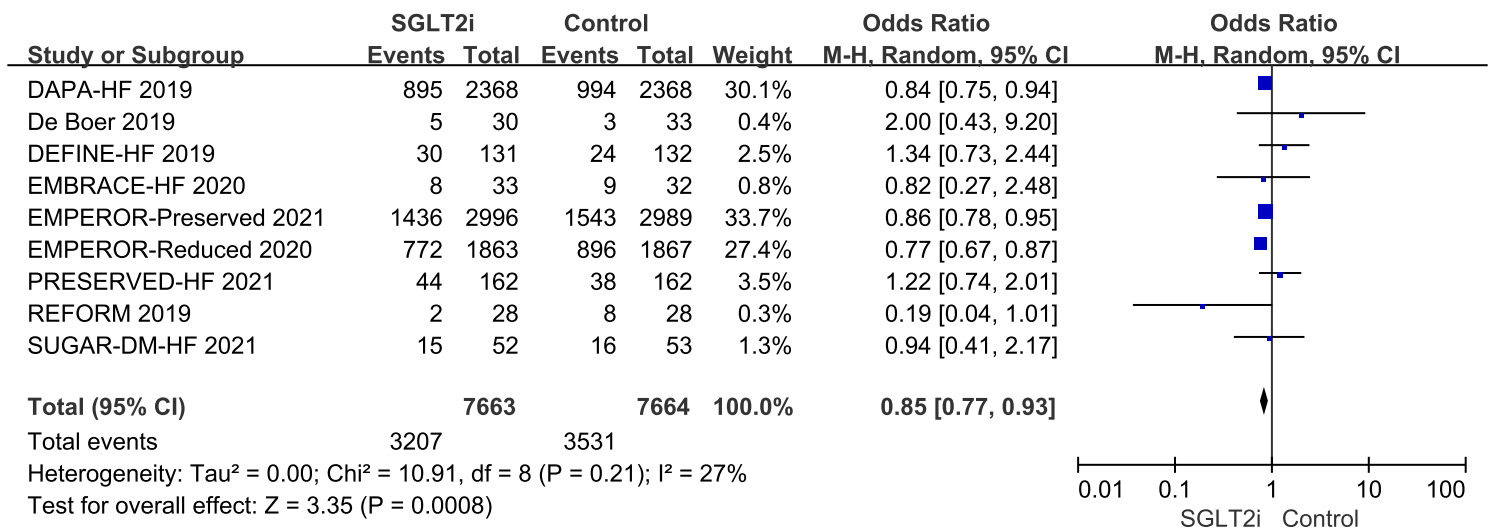

Supplement: Supplementary file 4 — Additional file 4: Figure S4. Forest plot showing the difference in serious adverse events between the SGLT2i groups and the control groups. CI, confidence interval; SD, standard deviation; SGLT2i, sodium-glucose cotransporter-2 inhibitors. [file 12933_2022_1574_MOESM4_ESM.pdf]

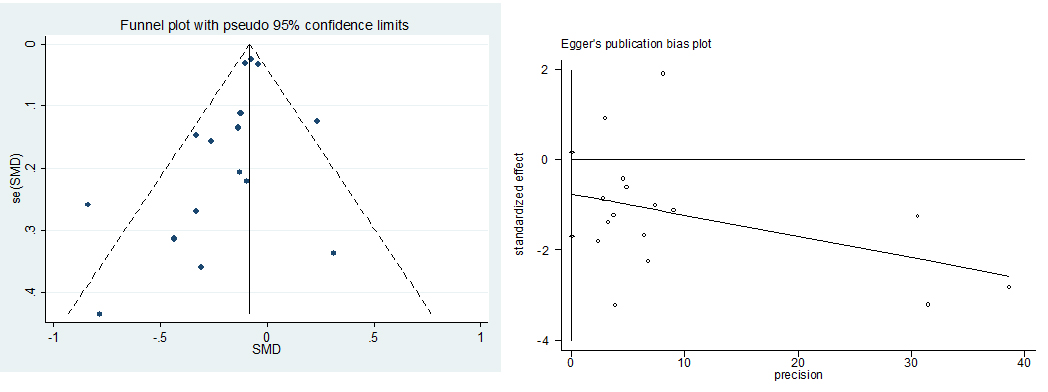

Supplement: Supplementary file 5 — Additional file 5: Figure S5. Funnel plot and Egger regression test for systolic blood pressure. SE, standard error; SMD, standard mean difference. [file 12933_2022_1574_MOESM5_ESM.jpg]
