# Supplementary material for: Effect of sodium-glucose cotransporter-2 inhibitors on blood pressure in patients with heart failure: a systematic review and meta-analysis
Source: Cardiovasc Diabetol. 2022 Jul 25;21:139. doi: 10.1186/s12933-022-01574-w (PMC9317067; doi:10.1186/s12933-022-01574-w)
Supplement: Supplementary file 6 — Additional file 6: Table S1. Baseline drugs and characteristics. Table S2. Sensitivity Analyses of the SGLT2i on SBP. Table S3. GRADE summary of findings. [file 12933_2022_1574_MOESM6_ESM.docx]

**Supplementary Table 1 Baseline drugs and characteristics**

| **Trial or Author** | **Age**  **（I/C）** | **Sex**  **Male**  **(I/C）** | **BMI**  **（I/C）** | **LVEF**  **（I/C）** | **NYHA≥III**  **(I/C）** | **Hypertension**  **(I/C）** | **Diabetes**  **(I/C)** | **ACEI/ARB**  **(I/C)** | **β-blocker**  **(I/C)** | **MRA**  **(I/C)** | **Diuretic**  **(I/C)** | **Insulin**  **(I/C)** | **Metformin**  **(I/C)** | **DPP-4 inhibitor**  **(I/C)** | **GLP-1R**  **A**  **(I/C)** |
| --- | --- | --- | --- | --- | --- | --- | --- | --- | --- | --- | --- | --- | --- | --- | --- |
| DAPA-HF [3] | 66.2±11.0  /66.5±10.8 | 76.2/77 | 28.2±6.0  /28.1±5.9 | 31.2±6.7  /30.9±6.9 | 32.3/32.7 |  | 41.8/41.8 | 84.5/82.8 | 96/96.2 | 71.5/70.6 | 93.4/93.5 | 27.6/26.9 | 50.8/51.7 | 16.2/15.1 | 1.1/1.0 |
| DEFINE-HF [19] | 62.2±11.0  /60.4±12.0 | 72.5/74.2 | 30.7 (27.3, 35.9)  /30.6(27.6,36.4) | 27.2±8.0  /25.7±8.2 | 30.5/  37.9 |  | 61.8/64.4 | 58.0/60.6 | 99.2/  93.9 | 58.0  /63.6 | 87.0/84.1 | 51.9/  52.9 | 35.8/38.8 | 13.6/11.8 | 4.9/1.2 |
| De Boer [31] | 68.5(62.0–74.0)  /71.0(59.0–74.0) | 66.7/57.6 | 31.2(28.8–34.7)  /31.3(28.4–34.2) | 53.9(45.4–63.7)/  55.4(43.4–61.8) | 26.7  /24.2 |  |  | 3.3/3.3 |  |  | 0/3.3 |  | 6.7 /21.2 | 0/3.0 |  |
| MUSCAT‐HF [30] | 71.7±7.7  /74.6±7.7 | 66/59 | 25.4±4.3  /25.3±4.4 | 57±9.4/58±9.4 | 4/1 | 89/79 |  | 61/57 | 64/57 | 23/24 | 23/23 |  |  |  |  |
| REFORM [20] | 66.9 ±7.0  /67.4 ±6.8 | 64.3/67.9 | 33.0±5.5  /32±5.2 | 44.5±12.4  /46.5 ±11.7 | 10.7  /14.3 | 78.6/64.3 | 6.5/6.17 | 89.3/89.3 | 85.7/78.6 | 46.4  /35.7 | 20.5/23.4 | 21.4/  35.7 | 60.7/50.0 |  |  |
| CANDLE [27] | 68.3 ± 9.8  /68.9 ± 10.4 | 77.9/71.7 | 24.1 ± 6.4  /25.4 ± 4.8 |  | 1.8/2.5 | 43.4/44.2 |  | 78.8/73.3 | 72.6/  68.3 | 37.2  /36.7 | 40.7/  44.2 | 3.5/2.5 | 15.9/21.7 | 56.6/52.5 | 0.9/0.8 |
| CANA-HF [28] | 58 ± 6.1  /54.3 ± 8.8 | 76.5/78.9 | 34.5 ± 6.6  /38.8 ± 7 | 31.6±7.5  /27.0 ± 6.8 | 41.2/36.8 | 94.1/89.5 |  | 82.4/57.9 | 94.1/  94.7 | 52.9  /68.4 | 76.5/94.7 | 64.7/31.6 | 58.8/52.6 | 0/5.3 | 5.9/0 |
| EMPEROR-Reduced [4] | 67.2±10.8  /66.5±11.2 | 76.5/75.6 | 28.0±5.5  /27.8±5.3 | 27.7±6.0  /27.2±6.1 | 24.9/25 | 72.4/72.3 | 49.8/49.8 | 88.8/89.6 | 94.7/ 94.7 | 70.1  /72.6 |  |  |  |  |  |
| EMPEROR-Preserved [22] | 71.8±9.3  /71.9±9.6 | 55.4/55.3 | 29.77±5.8  /29.90±5.9 | 54.3±8.8  /54.3±8.8 | 18.7/18.1 | 90.8  /90.4 | 48.9  /49.2 | 81  /80.4 | 86.7  /85.9 | 37.3  /37.6 |  |  |  |  |  |
| EMBRACE-HF [23] | 69.5±12.0  /62.9±13.3 | 63.6/62.5 | 33.5 (28.6,39.5)/  33.8 (29.4,40.0 | 46.7±14.9  /40.7±17.2 | 54.5/50 |  | 54.5  /50 | 33.3/25 | 87.9/90.6 | 27.3  /62.5 | 93.9/100 | 61.1/68.8 |  | 11.1/12.5 | 16.7/25 |
| RECEDE-CHF [18] | 69.8 ± 5.7 | 73.9 | 33.9 ± 5.6 |  |  |  |  | 87 | 87 | 47.8 | 100 | 21.7 | 69.6 |  |  |
| CANONICAL [29] | 76.5±6.4  /75.9±5.8 | 66.7/67.5 | 24.7±3.6  /25.2±3.7 | 61.1±7.8  /61.9±7.6 | 11.9/5 | 95.2/85 |  | 88.1/80 | 69.0/72.5 | 14.3/20 | 42.9/45 |  | 16.7/20 | 42.9/47.5 |  |
| Empire HF [24] | 65 ±10/  63 ±12 | 83/87 | 29(27-33)  /29(26-33) | 29±8/  30 ±8 | 19/12 | 37/43 | 12/14 | 62/68 | 96/94 | 65/66 | 66/65 |  |  |  |  |
| PRESERVED-HF [21] | 69 (64,77)  /71 (63, 78) | 43.2/43.2 | 35.1 (30.4, 41.8)/  34.6 (29.7,40.4) | 60 (55, 65)/  60 (54, 65) | 40.1/44/4 |  | 55.6/56.2 | 60.5/60.5 | 73.5/71.6 | 30.9/42 | 93.2/83.3 |  |  |  |  |
| Pietschner [25] | 69.0±8.1/  67.4±8.7 | 80.6/94.1 | 28.7±4.0  /29.2±3.3 | 39.8±8.3/  36.8±9.1 |  | 75/88.2 | 27.8/17.6 |  |  |  |  |  |  |  |  |
| SUGAR-DM-HF [26] | 68.2±11.7  /69.2±10.6 | 65.4/81.1 | 30.9 ±5.9  /30.4±5.1 | 32.1 ±10.3/  32.9±9.3 | 28.8/17 | 75/66 | 76.9  /79.2 | 94.2  /96.2 | 88.5/94.3 | 61.5  /58.5 | 59.6/54.7 | 19.4/10 | 80.6/70 | 41.9/30 |  |

Values are shown as the absolute number (percentage), median (interquartile range), or mean ± standard deviation. ACEI, angiotensin-converting enzyme inhibitor; ARB, angiotensin II receptor blocker; BMI, body mass index; DPP-4, dipeptidyl peptidase 4; GLP-1RA, glucagon-like peptide-1 receptor agonist; LVEF, left ventricular ejection fraction; MRA, mineralocorticoid receptor antagonist; NYHA, New York Heart Association.

**Supplementary Table 2 Sensitivity Analyses of the SGLT2i on SBP**

| **Study** | **MD 95CI** | **P** | **I^2^** |
| --- | --- | --- | --- |
| DAPA-HF [3] | -1.89 (-3.20, -0.58) | 0.005 | 48 |
| DEFINE-HF [19] | -1.80 (-2.67, -0.92) | <0.0001 | 31 |
| De Boer [31] | -1.67 (-2.71, -0.63) | 0.002 | 48 |
| MUSCAT‐HF [30] | -1.59 (-2.64, -0.54) | 0.02 | 47 |
| REFORM [20] | -1.64 (-2.66, -0.61) | 0.002 | 47 |
| CANDLE [27] | -1.70 (-2.79, -0.61) | 0.002 | 49 |
| CANA-HF [28] | -1.73 (-2.75, -0.72) | 0.0008 | 46 |
| EMPEROR-Reduced [4] | -1.99 (-3.21, -0.76) | 0.002 | 46 |
| EMPEROR-Preserved [22] | -1.98 (-3.32, -0.64) | 0.004 | 49 |
| EMBRACE-HF [23] | -1.37 (-2.14, -0.60) | 0.0005 | 23 |
| RECEDE-CHF [18] | -1.56 (-2.56, -0.56) | 0.002 | 44 |
| CANONICAL [29] | -1.71 (-2.76, -0.65) | 0.002 | 49 |
| Empire HF [24] | -1.50 (-2.51, -0.49) | 0.04 | 43 |
| PRESERVED-HF [21] | -1.69 (-2.77, -0.60) | 0.002 | 49 |
| Pietschner [25] | -1.63 (-2.66, -0.60) | 0.002 | 47 |
| SUGAR-DM-HF [26] | -1.70 (-2.76, -0.64) | 0.002 | 49 |

CI: confidence interval. MD: mean difference.

**Supplementary Table 3 GRADE summary of findings**

| **Outcomes** | **Pooled outcomes (95% CI)** | **No. of patients  (no.of included studies)** | **Statistical heterogeneity** | **Quality of evidence (GRADE)** |
| --- | --- | --- | --- | --- |
| Mean change in systolic blood pressure (mmHg) | MD -1.68 (-2.70, -0.66) | 15282 (16 studies) | I^2^=45% (P=0.03) | ⊕⊕⊕⊝ moderate^1^ |
| Mean change in diastolic blood pressure (mmHg) | MD -1.06 (-3.20, 1.08) | 751 (8 studies) | I^2^=43% (P=0.09) | ⊕⊕⊕⊝  moderate^1^ |
| Mean change in body weight (kg) | MD -1.36 (-1.68, -1.03) | 14539 (11 studies) | I^2^=61% (P=0.004) | ⊕⊕⊕⊝ moderate^1^ |
| Mean change in Hematocrit(%) | MD 1.63 (0.63, 2.62) | 14304 (9 studies) | I^2^=100% (P<0.001) | ⊕⊕⊝⊝  low^2^ |
| Mean change in HR (bpm) | MD -0.35 (-2.05, 1.35) | 702 (7 studies) | I^2^=0% (P=0.69) | ⊕⊕⊕⊕  high |
| Mean percentage change in HbA1c (%) | MD -0.16 (-0.28, -0.04) | 14057 (6 studies) | I^2^=91% (P<0.001) | ⊕⊝⊝⊝  very low^2,3^ |
| Mean change in NT-proBNP (pg/ml) | MD -60.31 (-105.43, -15.20) | 14870 (11 studies) | I^2^=77% (P<0.001) | ⊕⊕⊝⊝  low^2^ |
| Mean change in KCCQ | MD 1.97 (1.16, 2.77) | 14437 (7 studies) | I^2^=29% (P=0.21) | ⊕⊕⊕⊕  high |
| Mean change in eGFR (mL/min/1.73 m²) | MD 0.98 (-0.20, 2.17) | 10578 (10 studies) | I^2^=73%  (P<0.001) | ⊕⊕⊝⊝ low^2^ |

CI: confidence interval. MD: mean difference. eGFR: Estimated glomerular filtration rate. HbA1c: Glycated hemoglobin. HR: Heart rate. NT-proBNP, N-Terminal pro B-type Natriuretic Peptide. KCCQ: Kansas City Cardiomyopathy Questionnaire

GRADE Working Group grades of evidence

High quality: Further research is very unlikely to change our confidence in the estimate of effect.

Moderate quality: Further research is likely to have an important impact on our confidence in the estimate of effect and may change the estimate.

Low quality: Further research is very likely to have an important impact on our confidence in the estimate of effect and is likely to change the estimate.

Very low quality: We are very uncertain about the estimate.

^1^ Downgraded by one level for moderate statistical inconsistency (P <0.1)

^2^ Downgraded by two levels for severe statistical heterogeneity

^3^ Confidence interval crosses the non-effect value.
